# A Descriptive Comparative Analysis of the Surgical Management of Adrenal Tumors: The Open, Robotic, and Laparoscopic Approaches

**DOI:** 10.3389/fsurg.2022.848565

**Published:** 2022-03-03

**Authors:** Hassan Al-Thani, Noora Al-Thani, Maryam Al-Sulaiti, Abdelhakem Tabeb, Mohammad Asim, Ayman El-Menyar

**Affiliations:** ^1^Department of Surgery, Hamad General Hospital, Doha, Qatar; ^2^Department of Internal Medicine, Hamad General Hospital, Doha, Qatar; ^3^Department of Surgery, Clinical Research, Trauma and Vascular Surgery, Hamad General Hospital, Doha, Qatar; ^4^Department of Clinical Medicine, Weill Cornell Medical School, Doha, Qatar

**Keywords:** adrenal, laparoscopic, robotic, incidentalomas, open surgery, tumor

## Abstract

**Background:**

Currently, adrenalectomies are trending toward minimally invasive approach including robotic and laparoscopic surgery. We aimed to describe the clinical presentation and outcomes associated with the 3 different surgical approaches in patients who underwent adrenalectomy for adrenal mass at a single tertiary center.

**Methods:**

A retrospective descriptive observational study was conducted to include all patients who underwent surgical interventions for adrenal gland mass between 2004 and 2019. Patients were categorized into three groups according to the interventional approach (open, robotic vs. laparoscopic adrenalectomy) and data were analyzed and compared.

**Results:**

A total of 124 patients underwent adrenalectomies (61.3% robotic, 22.6% open, and 16.1% laparoscopic approach). Incidentally discovered adrenal mass was reported in 67% of patients, and hypertension was the most prevalent comorbidity (53%). The tendency for malignancy increased with increasing tumor size while the functioning tumors were more in the smaller tumor size. Larger tumors were more common in younger patients. The robotic approach showed shorter surgical intensive care and hospital length of stay. Patients in the open adrenalectomy group frequently presented with abdominal pain (*p* = 0.001), had more nonfunctional adrenal mass (*p* = 0.04), larger mean tumor size (*p* = 0.001), and were frequently operated on the right side (*p* = 0.03). There was no post-operative mortality; however, during follow-up, 8 patients died (3 open, 3 laparoscopic and, 2 robotic approach). The median follow-up was 746 days (range 7–5,840).

**Conclusions:**

The study explored the three surgical adrenalectomy approaches in a dedicated center for patients with adrenal pathology. It showed that robotic adrenalectomy could be safe and effective surgical approach for patients with benign functioning adrenal tumors of a diameter <6 cm. However, the choice of a surgical approach varies according to the adrenal mass presentation, patient fitness for surgery, type and sizes of the tumor, surgeon's experience, and hospital resources. Open surgery is considered the first choice for larger, ruptured adrenal tumor or malignancy. However, the recent restructuring of the surgical department resulted in selection bias in favor of the robotic surgery. Further studies are required to address the risk factors, selection criteria for appropriate management, cost, and quality of life.

## Introduction

The prevalence of adrenal masses has significantly increased over the past few decades along with the advanced diagnostic tools (1). Most of these masses are clinically silent and discovered incidentally upon imaging studies performed for other indications that are not related to adrenal diseases and therefore referred to as incidentalomas (2–4). Adrenal adenomas can be presented as unilateral or bilateral masses which may also develop due to metastasis from other primary cancer, infectious process, lymphoma, and neuroblastoma (5). In addition, some genetic conditions may increase the susceptibility for adrenal tumors (6–8).

Data on adrenal incidentalomas are often derived from autopsy studies which reported a prevalence rate of 1–8.7% depending on the age, tumor size, as well as the imaging tool (0.8–5%) (2, 3, 9). Notably, the tumor size may predict the risk of malignancy as patients with larger tumor sizes (>4 cm) are more likely to have a malignant disease and higher morbidity (10). Also, advanced age is a risk factor as the frequency of incidentalomas which are higher (7–10%) among the elderly as compared to the young adults (0.2%) (11, 12). The functionality of adrenal mass is another important aspect to be considered before planning the management. In some selective cases, surgical resection is the only possible option. Therefore, investigation of specific biochemical markers, selection of appropriate imaging modality, and tailored therapeutic and surgical interventions play an important role in the patient management and outcome.

Over the past few decades, the management of adrenal tumors has shifted from open radical adrenalectomy to advanced minimally invasive robotic and laparoscopic surgery (13). Notably, patients with uncomplicated adrenal tumors may have comparable outcomes with the laparoscopic as well as robotic surgical approaches (14). However, complex adrenal disease characterized by large tumor size (i.e., >8 cm), right-sided tumor, large phaeochromocytoma, or paraganglioma may have a better outcome with robotic surgery (15, 16). Moreover, a multidisciplinary team with proper communication between the anesthesiologist and the surgeon is important for safe surgical management (4). A previous study from our center revealed that one out of 100 patients who underwent abdominal computed tomography (CT) scanning had incidental findings of adrenal tumors (17). Of note, adrenal tumors require a better understanding of the management approach and its associated outcomes in our region in the Middle East. A recent review from the region showed that the total number of Robot-assisted surgery in the Middle East is still low and there are 19 da Vinci Surgical Systems installed in Saudi Arabia; 6 in Qatar; 3 in the United Arab Emirates; 2 in each of Kuwait and Lebanon; and only 1 in Egypt (18). Apart from the comparison of laparoscopic and open surgery in a small sample size, no published data is comparing the 3 surgical approaches for adrenal pathology from the Arab Middle East (19). The present study aims to describe the frequency, clinical presentation, and outcomes associated with the 3 surgical approaches in patients who underwent adrenalectomy (open, robotic, or laparoscopic) for adrenal pathology.

## Methods

A retrospective comparative observational study was conducted to include patients who had adrenal mass and presented to the general surgery department at Hamad General Hospital (HGH) between January 2004 and December 2019. All adult patients of any gender who underwent surgical interventions for adrenal mass were included in the study. Indication for surgical intervention based on the clinical presentation, functionality of the adrenal lesion (whether adenoma or hyperplasia), patient fitness, and the finding on CT imaging. Regarding the clinical presentation, all functional adrenal lesions referred from the endocrine clinics were offered a minimally invasive surgical approach in the form of laparoscopic or robotic-assisted surgery as the first option unless there is a contraindication. The robotic-assisted surgery (da Vinci surgical robot system) was installed at the HGH in 2009 and becomes the approach of choice. Laparoscopic surgery was introduced in 2005 in our hospital. Patients who presented with hypotension secondary to ruptured adrenal mass with hemorrhage underwent open transperitoneal adrenalectomy. The option of angioembolization for active bleeding was done on a selective basis depending on patients' general condition, availability of surgeon, and interventional radiologist. A benign lesion on a CT scan, especially the cystic one was mostly treated with minimum invasive surgery regardless of the tumor size. Any large lesions on the CT scan (>6 cm) or suspicious of malignancy were offered open surgery.

Retrieved data from the surgical database and electronic medical records included patients' demographics, clinical presentation, comorbidities, radiological findings, size and laterality of the adrenal pathology, functional status of the tumor, surgical approaches (robotic, open, and laparoscopic), operation time, blood loss and transfusion requirement (intra- and post-operative), complications, surgical intensive care unit (SICU) length of stay and total hospital stay, in-hospital mortality, follow-up outcome, and cause of death.

The American Society of Anesthesiologists (ASA) physical status classification system was used for assessing the fitness of patients before surgery.

The study was conducted in full conformance with principles of the “Declaration of Helsinki,” Good Clinical Practice (GCP), and within the laws and regulations of MoPH in Qatar. The Medical Research Center (MRC-01-20-254) at Hamad Medical Corporation has approved the study with a waiver of consent as de-identified data with no direct contact with the patients were collected retrospectively. This study follows the STROBE checklist of items that should be included in reports of observational studies (Supplementary Table).

### Statistical Analysis

Data were presented as frequency, mean ± standard deviation, and median and range, whenever appropriate. Patients were categorized into three groups according to the interventional approach, that is, open, robotic, or laparoscopic adrenalectomy. Differences in categorical variables were analyzed using χ^2^ test and Yates' corrected Chi-square if the expected cell frequencies were below 5. The continuous variables between different groups were compared using Student's *t*-test or one-way analysis of variance (ANOVA) test for more than 2 groups. Receiver Operating Characteristic (ROC) curve analysis was performed for the optimum tumor size cutoff, plotted against the open surgical intervention. Patient groups were also compared based on the different tumor size. The area under the curve (AUC) and the C-statistic were calculated to evaluate the performance and discriminatory power of the tumor size. Two-tailed *p* values < 0.05 were considered as significantly different. Data analysis was carried out using the Statistical Package for Social Sciences version 26 (SPSS Inc., Chicago, IL, United States).

## Results

During the study period, a total of 124 patients with adrenal mass underwent adrenalectomy. Table 1 represents the demographics, clinical presentation, comorbidities, management, and outcomes of the study cohort. The mean age of patients was 45.6 ± 12.4 years and 52% were males. Adrenal tumors were incidentally discovered in 67% of the patients. The modalities of diagnosis and ASA classification are shown in Table 1.

**Table 1:** Demographics, clinical presentation, management, and outcome of patients with adrenal mass underwent surgical treatment (*n* = 124).

**Variables**	**Value**	**Variables**	**Value**
Age	45.6 ± 12.4	**ASA classification (*****n** **=*** **119)**	
Males	65 (52.4%)	I	4 (3.4%)
Females	59 (47.6%)	II	74 (62.2%)
Qatari	25 (20.2%)	III	40 (33.6%)
Body mass index	29.1 ± 6.5	IV	1 (0.8%)
**Clinical presentation**		Extra-adrenal	5 (4.1%)
Incidental	83 (66.9%)	**Surgical approach**	
Abdominal pain	45 (36.3%)	Open adrenalectomy	28 (22.6%)
Fatigue	24 (19.4%)	Robotic adrenalectomy	76 (61.3%)
Muscle weakness/cramping	24 (19.4%)	Laparoscopic adrenalectomy	20 (16.1%)
Headache	28 (22.6%)	Additional procedure with adrenalectomy	8 (6.5%)
Palpitations	7 (5.6%)	**Any conversion**	2 (1.6%)
Back pain	14 (11.3%)	**Operation laterality**	
Weight loss	2 (1.6%)	Left	76 (61.2%)
Hirsutism	3 (2.4%)	Right	47 (38%)
Seizures	1 (0.8%)	Bilateral	1 (0.8%)
Spine stress fracture	1 (0.8%)	**SICU admission**	34 (27.4%)
Multiple endocrine neoplasia type 1 (MEN1).	3 (2.4%)	SICU days	2 (1-6)
Previous abdominal surgery	22 (17.7%)	**Blood loss (ml)**	100 (20-5250)
History of other malignancy	9 (7.3%)	**Transfusion**	16 (12.9%)
Co-morbidities		Intra-operative	16 (12.9%)
Hypertension	66 (53.2%)	Post-operative	2 (1.6%)
Diabetes	24 (19.4%)	**Operation time**	185.6 ± 68.8
Coronary artery disease	6 (4.8%)	**Length of hospital stay (days)**	5 (2-36)
Hypotension	5 (4.0%)	**Duration of follow-up (days)**	746 (range 7-5840)
Radiological investigations		**Post-operative mortality**	0 (0.0%)
CT scan	113 (91.1%)	**Death during follow-up**	8 (6.4%)
MRI	53 (42.7%)	**Cause of death**	
PET CT scan	8 (6.5%)	Cardiac arrest	2 (25%)
MIBG scan, iodine-131-meta-iodobenzylguanidine	2 (1.6%)	Advance-adrenal carcinoma/metastasis	3 (37.5%)
Pre-operative biopsy	7 (5.6%)	Hemorrhagic shock	1 (12.5%)
Pre-operative embolization	3 (2.4%)	Advance breast carcinoma	1 (12.5%)
Functional adrenal mass	66 (53%)	Advance colon cancer	1 (12.5%)
Nonfunctional adrenal mass	58 (47%)		
Tumor size, cm	7.04 ± 5.1 (range 0.6–30)		

**Figure 1:**
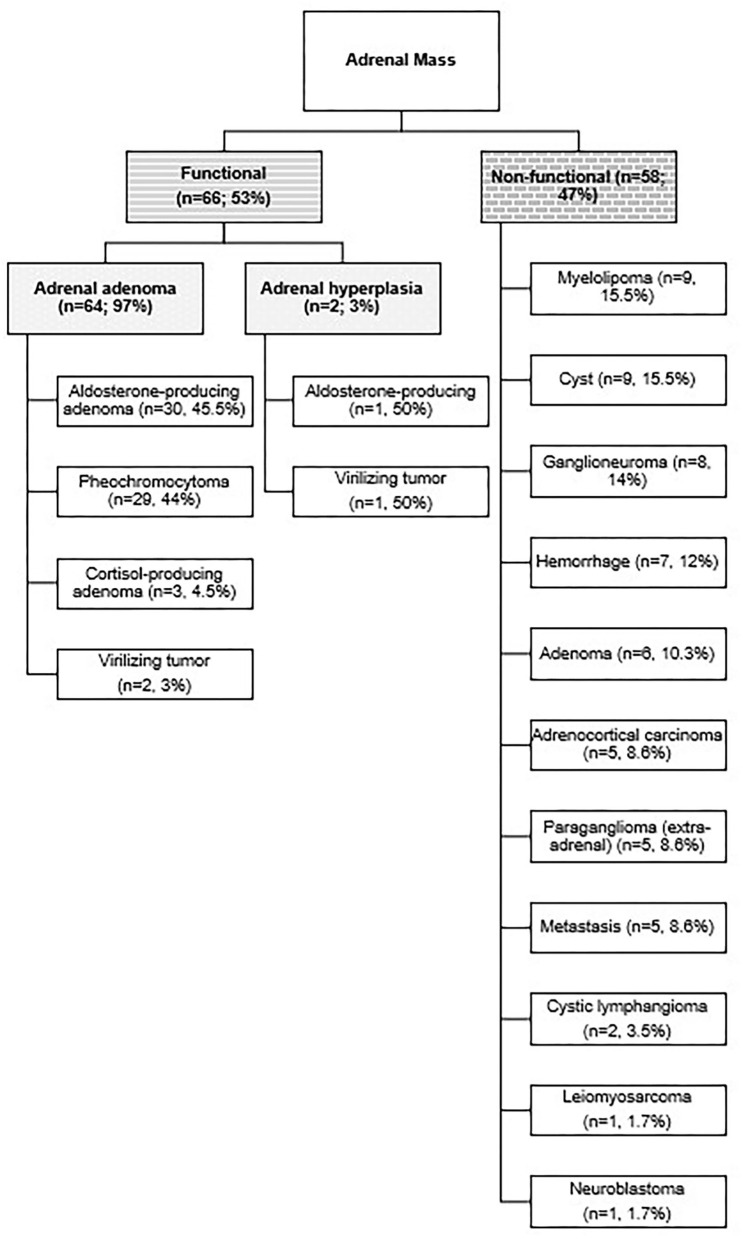
The distribution of adrenal mass in terms of functionality and hormone production.

Figure 1 shows the nature of the adrenal mass in terms of functionality and hormone production. The mean tumor size was 7.04 ± 5.1 cm. The median tumor size was 3.5 cm (range 0.6–30), 8.0 cm (range 1.5–19), and 9.0 (4.5–30) in the functioning, non-functioning, and malignant masses, respectively.

Robotic adrenalectomy (61.3%) was the most frequent surgical approach followed by open (22.6%) and laparoscopic adrenalectomy (16%). Eight (6.5%) patients had additional procedures along with adrenalectomy and two (1.6%) had a conversion. About 61% of patients had left-sided, 38% had right-sided operation, and one patient was operated for bilateral tumor. Adrenal carcinoma was discovered in 5 patients and metastasis from extra-adrenal cancer was found in another 5 patients. There were 2 cases that required conversion to open surgery due to bleeding from the adrenal vein during the early phase of the adoption of robotic surgery, but it did not impact the patients' s outcome.

Table 2 compares the demographics, clinical presentation, and hospital course based on the surgical approach. Patients in the laparoscopic adrenalectomy group were more likely to be older (*p* = 0.04), had a higher frequency of ASA classification III (*p* = 0.001), and frequently underwent left-sided operations (*p* = 0.03) as compared to the other groups. The significantly higher proportion of patients with hypertension (*p* = 0.01) and ASA classification II (*p* = 0.001) had robotic adrenalectomy and this group had a smaller mean tumor size (4.9 ± 2.7 cm) than the other groups. Patients in the open adrenalectomy group were frequently presented with abdominal pain (*p* = 0.001), had more non-functional adrenal mass (*p* = 0.04), larger mean tumor size (*p* = 0.001), frequently were operated on the right side (*p* = 0.03), and had prolonged SICU stay (*p* = 0.001).

**Table 2:** Demographics, clinical presentation, and hospital course based on the surgical approach.

	**Open** **(*n =* 28)**	**Robotic** **(*n =* 76)**	**Laparoscopic (*n =* 20)**	***P*-value**
Age	40.9 ± 9.4	46.3 ± 12.8	49.4 ± 13.4	0.04
Males	17 (60.7%)	40 (52.6%)	8 (40.0%)	0.36 for all
Females	11 (39.3%)	36 (47.4%)	12 (60.0%)	
Body mass index	26.9 ± 6.7	29.7 ± 6.4	29.6 ± 7.0	0.20
Qatari patient	1 (3.6%)	15 (19.7%)	9 (45.0%)	0.002
Incidental finding	20 (71.4%)	52 (68.4%)	11 (55.0%)	0.44
Abdominal pain	19 (67.9%)	23 (30.3%)	3 (15.0%)	0.001
Palpitation	1 (3.6%)	5 (6.6%)	1 (5.0%)	0.83
Hypertension	8 (28.6%)	47 (61.8%)	11 (55.0%)	0.01
Diabetes mellitus	5 (17.9%)	17 (22.4%)	2 (10.0%)	0.44
Functional adrenal mass	10 (35.7%)	44 (57.9%)	12 (60.0%)	0.10
Nonfunctional adrenal mass	19 (67.9%)	31 (40.8%)	8 (40.0%)	0.04
Tumor size (cm)	13.4 ± 5.2	4.8 ± 2.8	5.1 ± 3.4	0.001
**ASA classification (*****n** **=*** **119)**				
I	1 (3.6%)	3 (3.9%)	0 (0.0%)	0.001 for all
II	15 (53.6%)	56 (73.7%)	3 (20.0%)	
III	12 (42.9%)	17 (22.4%)	11 (73.3%)	
IV	0 (0.0%)	0 (0.0%)	1 (6.7%)	
Pre-operative biopsy	3 (10.7%)	3 (3.9%)	1 (5.0%)	0.41
Pre-operative embolization	2 (7.1%)	1 (1.3%)	0 (0.0%)	0.17
Additional procedure with adrenalectomy	1 (3.6%)	6 (7.9%)	1 (5.0%)	0.69
Any conversion	0 (0.0%)	2 (2.6%)	0 (0.0%)	0.52
**Operation laterality**				
Left	13 (46.4%)	46 (60.5%)	17 (85.0%)	0.03 for all
Right	14 (50.0%)	30 (39.5%)	3 (15.0%)	
Bilateral	1 (3.6%)	0 (0.0%)	0 (0.0%)	
SICU admission	14 (50.0%)	17 (22.4%)	3 (15.0%)	0.008
SICU days	2.5 (1–6)	1 (1–2)	2 (1–3)	0.001
Blood loss (ml)	700 (50–5250)	100 (20–2000)	150 (50–750)	0.001
Intra-operative Transfusion	12 (42.9%)	2 (2.6%)	2 (10.0%)	0.001
Post-operative Transfusion	2 (7.1%)	0 (0.0%)	0 (0.0%)	0.03
Operative time	164 (60–340)	174 (60–385)	165 (105–295)	0.62

**Figure 2:**
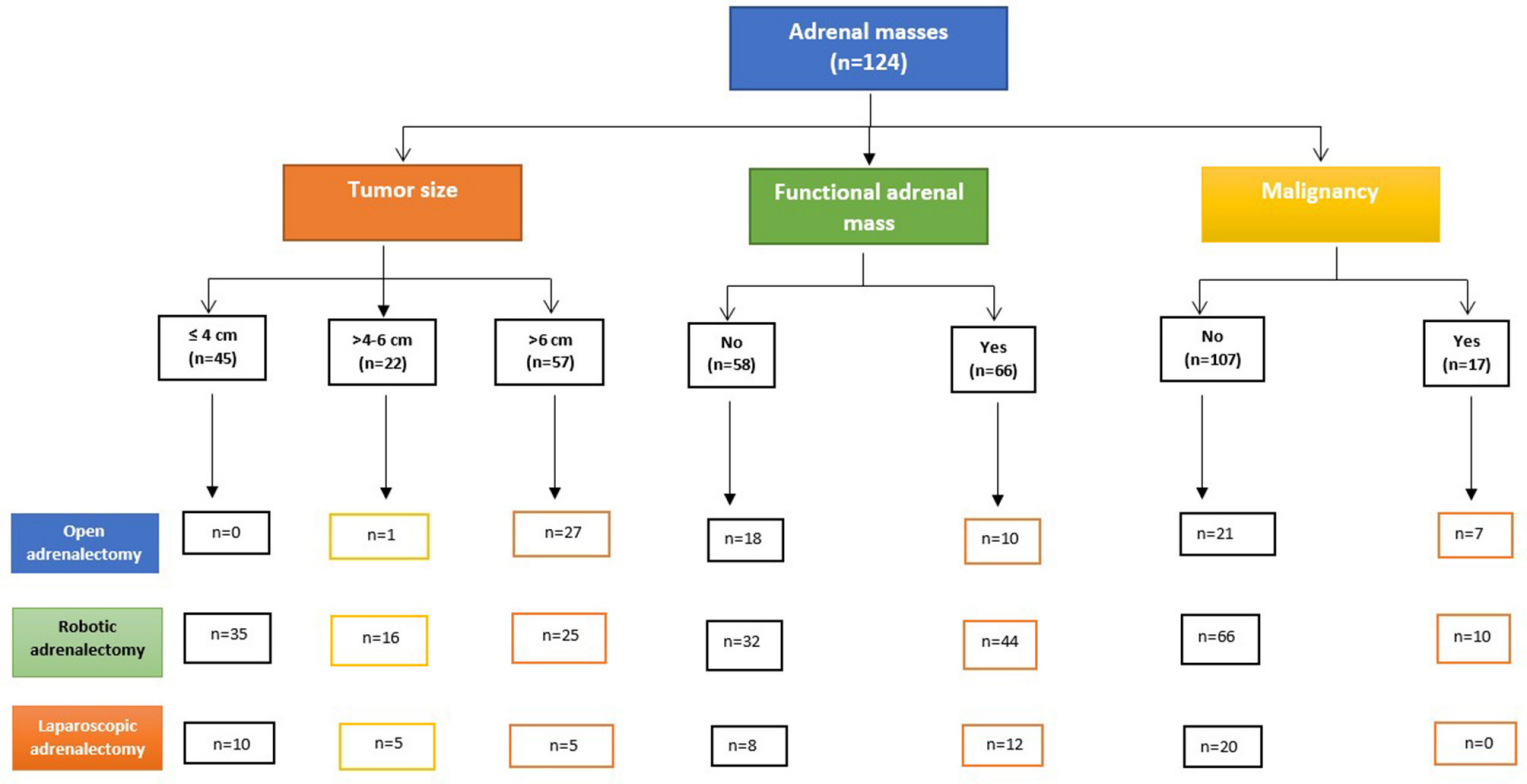
Study design for surgical approaches based on tumor size, functionality, and malignancy.

Table 3 and Figure 2 show the surgical approaches based on the tumor size. The tendency for malignancy increased with increasing the tumor size while the functioning tumors were more in the smaller tumor size. Larger tumors were more in younger patients. Gender distribution, pre-operative angioembolization, and operative time were comparable for all the groups. The open group had the highest median blood loss (*p* = 0.001) and transfusion as compared to the other groups.

**Table 3:** Clinical characteristics and surgical approach based on tumor size.

	**Tumor size**			
	**≤4 cm** **(*n =* 45, 36%)**	**>4–6 cm** **(*n =* 22; 18%)**	**>6 cm** **(*n =* 57; 46%)**	***P*-value**
Age (mean ± SD)	49.1 ± 13.1	46.9 ± 10.5	42.3 ± 11.9	0.01
**ASA classification (*****n** **=*** **119)**				
I–II	27 (65.9%)	14 (66.7%)	37 (64.9%)	0.988 for all
III–IV	14 (34.1%)	7 (33.3%)	20 (35.1%)	
**Surgical approach**				
Open adrenalectomy	0 (0.0%)	1 (4.5%)	27 (47.4%)	0.001 for all
Robotic adrenalectomy	35 (77.8%)	16 (72.7%)	25 (43.9%)	
Laparoscopic adrenalectomy	10 (22.2%)	5 (22.7%)	5 (8.8%)	
Functional adrenal mass	37 (82.2%)	11 (50.0%)	18 (31.6%)	0.001
Malignancy	0 (0.0%)	1 (4.5%)	16 (28.1%)	0.001

**Figure 3:**
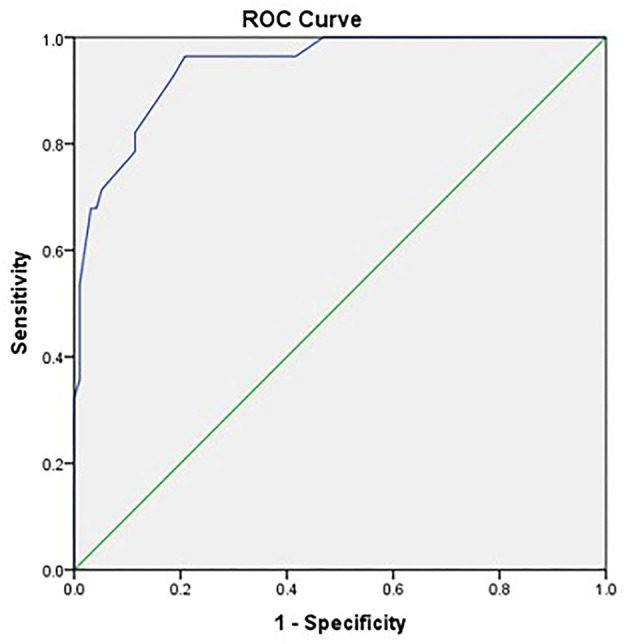
The receiver operating characteristic curve for tumor size cutoff to predict the use of open surgery rather than non-open surgical approaches (robotic or laparoscopic). Area under the curve is 0.94 (95% confidence interval: 0.902–0.986), *p* = 0.001.

The ROC curve to define the optimum tumor size for open surgery showed that tumor size greater than 4 cm had an AUC of 0.94 (95% CI 0.902–0.986) with sensitivity and specificity of 100 and 47%, respectively (Figure 3 and Table 4).

**Table 4:** Sensitivity and specificity of different tumor size (TS) cutoff in favor of open surgery (vs. non-open surgical approaches).

	**TS >4 cm**	**TS >5 cm**	**TS >6 cm**
Sensitivity	100%	96.4%	96.4%
Specificity	47%	58.3%	69%

Table 5 shows the in-hospital complications, hospital course, mortality, and follow-up. Seven patients developed complications which included bleeding from the adrenal vein (2 cases), post-operative pneumonia (2 cases), diaphragmatic injury (1 case), pleural effusion (1 case), and renal artery occlusion (1 case).

**Table 5:** Complications and outcome based on the surgical approach.

	**Open (*n =* 28)**	**Robotic** **(*n =* 76)**	**Laparoscopic (*n =* 20)**	***P*-value**
Complications (*n =* 119)	1 (3.7%)	4 (8.0%)	2 (11.8%)	0.59
	Renal artery occlusion secondary to pre-operative angio- embolization for rupture adrenal tumor with bleeding	2 cases Bleeding from the adrenal vein required conversion to open surgery. 1 case of Pleural effusion required drainage, 1 case of post-operative pneumonia	1 case of diaphragmatic injury repaired, and chest tube inserted, 1 case of post-operative pneumonia and abdominal collection	
Length of hospital stay days (median and range)	7 (2–22)	4 (2–29)	9 (3–36)	0.001
Death during follow-up	3 (10.7%)	2 (2.6%)	3 (15.0%)	0.25
Duration of follow-up (median and range) days	224 (13–3,832)	692.5 (7–4,708)	5,292 (1,451–5,840)	0.001

The length of stay in the SICU was significantly lower in patients with robotic surgery in comparison to other procedures. The median follow-up period was significantly higher for the laparoscopic group (*p* = 0.001) than the other groups. Eight patients died during the follow-up period (3 open, 3 laparoscopic, and 2 had robotic surgery). No mortality was directly related to the operation as all the deaths occurred after 465 days (range 465–5,548 days). The cause of death was cardiac arrest (*n* = 2), advance adrenal carcinoma metastasis (*n* = 3), hemorrhagic shock (*n* = 1), advanced breast (*n* = 1), and colon (*n* = 1) cancer.

## Discussion

This is primarily an observational comparative study of managing patients with adrenal pathology in a dedicated center. Adrenal gland surgery has progressed over the past few decades but is still a challenging procedure that needs to be performed by specialized surgeons. The three surgical approaches for adrenalectomy (open, robotic, and laparoscopic) should be tailored based on criteria that vary according to the adrenal pathology presentation, patient fitness, surgeon skills, and hospital resources. The present study presents our local experience in the surgical management of the adrenal pathology and the changes that had happened during the past few years. The HGH is the largest teaching hospital in Qatar, with Accreditation Council for Graduate Medical Education–International (ACGME-I) accredited surgical programs. All the operations are used as an opportunity to teach the residents and fellows the operative techniques which may impact the overall operative time. During the study period, there was a restructure of the surgical department resulting in subspecialty development. This had resulted in streamlining the adrenalectomy to two surgeons (one from the general surgery and one from the urology department; both had experience in robotic-assisted surgery). All the open adrenalectomy was done by the general surgeon, whereas the robotic approach was done by both the general and urology surgeons. This led to the wide adoption of the robotic adrenalectomy approach at the HGH. The present study is unique from an Arab Middle Eastern country to analyze the clinical characteristics and outcomes of patients with adrenal tumors treated with different surgical approaches. In addition to the long follow-up, the present study has several key findings. Incidental detection of adrenal mass was frequent, but its proportion was comparable among the different study groups. The study showed that robotic surgery is feasible in most of the adrenalectomies of variable sizes. Large tumor size (>6 cm) were removed by open (47%), robotic (44%), or laparoscopic (9%) approach. Small tumor size (<4 cm) were removed by robotic (78%) or laparoscopic (22%) approach. However, 73% of the intermediate-sized tumors (4–6 cm) were removed by robotic approach as it has better handling and manipulation of the tumor than the laparoscopic approach. Although ROC and AUC showed that tumor size greater than 4 cm is an indicator for open surgery, other factors should be considered such as tumor functionality, malignancy, surgeon experience, and physical fitness of the patient. A higher proportion of patients with severe systemic disease and not expected to survive without the operation, as per the ASA classification, underwent laparoscopic adrenalectomy. Patients who had open surgery were more likely to be admitted to the SICU with a longer length of stay. The longer intensive care length of stay and increased rate of bleeding in the open surgery group could be related to the presence of larger and right-sided tumors.

However, prolonged hospital stay was observed in patients treated with laparoscopic approach as well. This may reflect the tendency to adopt more robotic surgery in fitter patients as a consequence of the restructuring of our surgical department. Although there was no post-operative mortality in the 3 groups, patients in the laparoscopic group were more likely to die during the follow-up. However, all deaths that occurred during the follow-up were not related to any of the surgical approaches.

Notably, adrenal incidentalomas are common especially with the advanced age (20). A pool of 25 studies which included 87,065 autopsies showed that the prevalence of adrenal adenomas was 5.9% (range 1.1–32%) (21). Also, the findings of abdominal CT scan revealed a prevalence of adrenal incidentaloma (4%) (3). In the present study, all patients with incidentaloma underwent a thorough evaluation to assess the functionality and susceptibility for malignancy before surgery, as per the guidelines of the American Association of Clinical Endocrinology (AACE) (22).

Evidence suggests that larger tumor size (>4 cm) is a predictor of malignancy, and it is recommended to resect such tumors irrespective of the functional status (22, 23). In the present study, the average tumor size was 7 cm (0.6–30 cm) and patients with larger tumor sizes mostly underwent open adrenalectomy. Consistent with our findings, it has been suggested that conventional open technique should be considered for adrenal masses greater than 6 cm or suspected for malignancy upon radiological imaging (24).

A comparative study between robotic and laparoscopic resection of large adrenal tumors concluded that robotic surgery had shorter operative time and less conversion than laparoscopic resection (25). Notably, the tumor size was comparable in the 2 intervention groups. Another study by Zhu et al. (26) compared open vs. retroperitoneal laparoscopic adrenalectomy for patients with large pheochromocytoma (range 5–10 cm). The authors suggested that laparoscopic adrenalectomy could be replaced by open surgery in patients with tumor size >5 cm. Moreover, the retroperitoneal laparoscopic group had a shorter operative time, quicker bowel recovery, and shorter hospital length of stay in comparison to the open adrenalectomy group (26). However, this study had no giant tumors. Contrarily, in our study, open surgery was preferred for larger tumor size to overcome the technical difficulties. One patient had a tumor size of 30 cm which turned out to be adrenocortical carcinoma (ACC). Seven patients underwent preoperative percutaneous biopsy, as recommended by the AACE (22). Percutaneous fine-needle aspiration (FNA) is recommended for patients known to have primary carcinoma to confirm the pathology of the adrenal mass. However, it is not recommended for suspected cases for ACC due to the risk of tumor seeding along the needle pathway (22). A systemic review of 32 studies addressing indication and yield of adrenal biopsy concluded that FNA is preferred in patients with extra-adrenal tumors but not recommended for diagnosis of adrenocortical carcinoma (27). Adrenal tumors can be rich in vascularity and may cause significant blooding and so adrenal artery embolization is indicated to overcome this complication. In our study, 3 patients had pre-operative embolization; 2 had open surgery, and one underwent robotic surgery. Sormaz et al. (28) reported 3 cases of perioperative embolization for giant tumors (ranging 8–17cm), which revealed that this intervention could reduce the intraoperative blood loss.

In our study, one patient had bilateral open adrenalectomy and two patients from the robotic surgery group converted to open surgery secondary to bleeding from the adrenal vein. This lower rate of conversion is in line with the previous reports. Agcaoglue et al. (25) reported 1 conversion in the robotic group and 4 in the laparoscopic group. Aliyev et al. (16) compared laparoscopic and robotic interventions for patients with pheochromocytoma; 1 out of 25 patients in the robotic group and 3 out of 40 in the robotic group were converted to open surgery. In our study, the average body mass index (BMI) was 29.1 ± 6.5 and it did not differ significantly among the 3 groups. Like our findings, Aksoy et al. (29) reported no significant differences between the laparoscopic and robotic groups in patients with BMI ≤ 30 and > 30 kg/m^2^.

Colvin et al. (30) prospectively reviewed 20 robotic surgery and 16 laparoscopic surgery cases and found no difference in the hospital length of stay in the two groups. Zhu et al. (26) reported a shorter length of hospital stay in the laparoscopic group in comparison to the open surgery group. On the contrary, our study showed prolonged hospital stay in the laparoscopic adrenalectomy group than the open group. Notably, 80% of patients in the laparoscopic group had ASA III (severe systemic disease) or IV (a severe systemic disease that has a threat to life) which may attribute to the observed differences. A systematic review including 27 studies found that the robotic approach had a significantly shorter hospital stay and longer operating time compared to the laparoscopic approach; however, both approaches had similar clinical outcomes in the selected set of patients (31). A meta-analysis including 9 studies (32) showed that the operative times were similar, with no difference in conversion rates or complication rates, but a significantly higher estimated blood loss and hospital stay were identified in the laparoscopic group compared with the robotic group. Notably, the laparoscopic group patients were more obese (32).

In our series, open surgery resulted in more blood loss followed by laparoscopic and robotic surgery, and the need for intraoperative and post-operative blood transfusion was also higher in the open surgery group. Brandao et al. (33) reported that 1 out of 30 patients in the robotic group had intraoperative and 2 had post-operative transfusions; whereas 4 out of 46 patients in the laparoscopic group had intraoperative and 2 had postoperative transfusions. In our study, one patient in the open intervention group had renal artery occlusion secondary to preoperative angioembolization for rupture adrenal tumor with bleeding. Diaphragmatic injury and abdominal collection were reported in the laparoscopic group, and the robotic group had bleeding from the adrenal vein in 2 patients-−1 had post-operative pneumonia and 1 had pleural effusion. Consistent with our findings, an earlier study reported similar complications (34). In our study, none of the patients died post-operatively; however, during clinical follow-up, 8 patients died. An earlier study reported lower 30-day mortality (0.5%) post-laparoscopic adrenalectomy (35). A recent systematic review (36) including 17 studies showed that although open surgery is the gold standard approach, there were no significant differences observed in the rate and time to recurrence and cancer-specific mortality between laparoscopic and open approaches (especially for tumor stage I-II). Although the robotic approach has several advantages compared to the laparoscopic, the specific indications of robotic approach in adrenal carcinoma remain unclear (36). The perioperative safety was found to be similar in the laparoscopic and robotic approaches; however, the hospital length of stay was better in the robotic group in a meta-analysis of 21 studies (37).

## Limitations

The retrospective design is one of the limitations of the present study. Being single center, the frequency of patients who underwent adrenalectomy in different groups in our study are higher as compared to other published studies. Internal and external validation of the study findings would be of value in prospective and multicenter studies in the region. Our hospital is the only tertiary government not-for-profit facility in the country. In addition, most of the earlier studies mainly focused on two surgical approaches while we compared the three surgical intervention groups. The follow-up period was long and was justified to come up with conclusions about the immediate and long-term complications of the 3 approaches. Also, the present study did not address information regarding the hospital and surgeon volume, experience, and skill of surgeons as it could relate to the patient's outcomes. However prior data showed that surgical sub-specialty did not significantly influence the perioperative results, but the learning curve and volume workload play an important role in the outcomes (14). Selection bias cannot be ignored as the compared groups in the study did not adjust for some factors such as the comorbidities, disease under treatment, and grade of anesthesia. To minimize the selection bias in terms of the surgical approach, we adopted the European Society of Endocrine Surgeons recommendations for the surgical management of adrenocortical carcinoma. Minimally invasive surgery was performed for Stage I or II with a diameter <10 cm (38). It appears that there is an immediate adoption of the robotic platform for adrenalectomy, whereas some literature suggests a more gradual trend in the use of the robotic platform. Of note, the robotic approach has continued to increase among surgical procedures and hospitals that have adopted robotic surgery programs had an immediate and diffuse increase in robotic surgery with a relative decrease in the laparoscopic approach (39). Generally, open adrenalectomies were done for emergency cases presented with bleeding or large adrenal mass suspected to be adrenocortical carcinoma.

## Conclusions

The study explored the three surgical adrenalectomy approaches in a dedicated center for patients with adrenal pathology. It showed that robotic adrenalectomy could be safe and effective surgical approach in patients with benign functioning adrenal tumors of a diameter less than <6 cm. However, the choice of surgical intervention depends on the case presentation, patient fitness for surgery, type and sizes of the tumor, surgeon's experience, and hospital resources. Open surgery is considered the first choice for larger, ruptured adrenal tumor, or malignancy. However, the recent restructuring of the surgical department resulted in selection bias in favor of robotic surgery. Further studies are required to address the risk factors, selection criteria for appropriate management, and impact on cost and quality of life.

## Data Availability Statement

All data had been shown in the results section, tables, and figures. Access to raw data will need agreement from the medical research center at HMC, Doha (mrchelpdesk@hamad.qa).

## Ethics Statement

The study was conducted in full conformance with principles of the “Declaration of Helsinki”, Good Clinical Practice (GCP), and within the laws and regulations of MoPH in Qatar. The Medical Research Center (MRC-01-20-254) at Hamad Medical Corporation has approved the study with a waiver of consent as de-identified data with no direct contact with the patients were collected retrospectively.

## Author Contributions

HA-T and NA-T: conceptualization, study design, and manuscript writing and review. MA-S and AT: manuscript review. MA and AE-M: data analysis, manuscript draft, and review. HA-T, NA-T, MA-S, and AT: data collection. MA-S, MA, AE-M, and AT: interpretation. All authors have a substantial contribution in the study and approved the manuscript submission.

## Conflict of Interest

The authors declare that the research was conducted in the absence of any commercial or financial relationships that could be construed as a potential conflict of interest.

## Publisher's Note

All claims expressed in this article are solely those of the authors and do not necessarily represent those of their affiliated organizations, or those of the publisher, the editors and the reviewers. Any product that may be evaluated in this article, or claim that may be made by its manufacturer, is not guaranteed or endorsed by the publisher.
